# Comparison of quadriceps femoris properties, surface electromyography parameters and foot posture asymmetries between patients with unilateral and bilateral knee osteoarthritis

**DOI:** 10.3389/fphys.2025.1710819

**Published:** 2025-12-02

**Authors:** Xingxing Shen, Jiaqing Tian, Jiahao Chen, Jiahao Zhang, Sirun Cheng, Ruian Xiang, Xuemeng Xu

**Affiliations:** 1 The Fifth Clinical Medical School, Guangzhou University of Chinese Medicine, Guangzhou, Guangdong, China; 2 Guangdong Second Traditional Chinese Medicine Hospital, Guangzhou, Guangdong, China; 3 Guangdong Provincial Key Laboratory of Research and Development in Traditional Chinese Medicine, Guangzhou, Guangdong, China

**Keywords:** knee osteoarthritis, quadriceps femoris, surface electromyography, biomechanics, FPI-6, foot posture, asymmetry

## Abstract

**Objective:**

To investigate the differences in quadriceps femoris (QF) properties, surface electromyography (sEMG) parameters and foot posture asymmetries between patients with unilateral and bilateral knee osteoarthritis (KOA), and to analyze the factors related to foot posture asymmetry.

**Methods:**

A total of 32 patients with unilateral KOA (unilateral group, UG) and 35 patients with bilateral KOA (bilateral group, BG) were enrolled in this study. The severity of knee osteoarthritis symptoms was assessed, and the affected legs were categorized as relatively severe leg (RSL) or relatively moderate leg (RML) based on the Visual Analogue Scale (VAS). Surface electromyography was utilized to measure the root mean square (RMS) values of rectus femoris (RF), vastus medialis (VM), and vastus lateralis (VL) during a straight leg raise task. Biomechanical characteristics, including muscle tone and stiffness, were measured using MyotonPRO. The foot posture index-6 (FPI-6) was applied to assess foot posture and asymmetrical foot posture scores. Additionally, we calculated the asymmetry indices of muscle tone (Asy_Tone_), stiffness (Asy_Stiffness_), and root mean square (Asy_RMS_) for the QF, along with their FPI asymmetry scores.

**Result:**

In the evaluation of RF, VM, and VL in both groups, the RMS on the RML was significantly higher than that on the RSL (*P* < 0.05), while muscle tone and stiffness on the RSL were significantly higher than those of the RML (*P* < 0.05). In UG, Asy_Tone_ (RF), Asy_Tone_ (VM), Asy_Stiffness_ (RF), Asy_Stiffness_ (VM), Asy_Stiffness_ (VL) and Asy_RMS_ (VM) were significantly higher than those in BG (*P* < 0.05). Asy_Tone_ (VL) was significantly lower than that in BG (*P* < 0.01). There was no significant difference in Asy_RMS_ (RF) and Asy_RMS_ (VL) between the two groups (*P* > 0.05). Regarding the FPI asymmetry scores, the proportion of asymmetry (including asymmetry and severe asymmetry) in the UG (65.6%) was much more frequent compared with that of BG (34.3%), with a statistically significant difference (χ^2^ = 6.57, *P* = 0.01). Furthermore, the VAS score and K/L grade were significantly correlated with the FPI asymmetric score in the UG (*b* = 1.065; 95% CI: 0.194, 1.936; *p* = 0.019 and *b* = 1.770; 95% CI: 0.215, 3.325; *p* = 0.028, respectively) and BG (*b* = 0.665; 95% CI: 0.117, 1.212; *p* = 0.020 and *b* = 1.523; 95% CI: 0.414, 2.632; *p* = 0.009, respectively).

**Conclusion:**

Both unilateral and bilateral patients with KOA exhibited a propensity for asymmetry in the properties of the QF, RMS values, and foot postures on both sides. Notably, unilateral patients tended to demonstrate this asymmetry more prominently and exhibit a higher prevalence of foot posture asymmetry compared to those bilateral patients. Furthermore, the degree of foot posture asymmetry was closely linked to pain severity and K/L grading whether in unilateral or bilateral KOA patients.

## Introduction

Knee Osteoarthritis (KOA) is a type of chronic disease involving joints and surrounding tissues, which seriously affects patients’ work capacity and quality of life (Hunter and Bierma-Zeinstra, 2019). According to epidemiological surveys, approximately 27 million adults in America and around 250 million people worldwide suffer from KOA (Mora et al., 2018). The prevalence of KOA continues to rise with the aging of the population, imposing substantial economic burdens on individuals and society alike (Wallace et al., 2017). In order to solve this global health problem, it is necessary to find the pathogenesis of KOA for its prevention and treatment. As a multi-joint disease, KOA has been found to be associated with biomechanical changes in adjacent joints and muscles. Abnormal alterations in foot posture and biomechanical changes of the quadriceps femoris (QF) may be significantly associated with KOA (Gonçalves et al., 2017; Chen et al., 2023).

Abnormal foot posture, pronation, and supination in patients with KOA can affect the distribution of force in the lower limbs (Al-Bayati et al., 2018). The ankle serves as a pivotal point for lower limb power and deviations in ankle position can lead to biomechanical changes in the knee, hip, spine, and even body center of gravity (Resende et al., 2016). Therefore, it is necessary to assess foot posture for understanding KOA pathogenesis and formulating appropriate treatment measures. The Foot Posture Index-6 (FPI-6) is considered a rapid, direct, and cost-effective method for assessing foot posture, as it can effectively evaluate foot posture and categorize feet into three types: pronated, neutral, and supinated (Redmond et al., 2006). In addition, FPI-6 has higher reliability in patients with knee osteoarthritis compared with conventional measures (Evans et al., 2003). Previous studies have shown that foot posture evaluated by FPI in KOA patients was more varus than that of healthy subjects, and foot posture was closely related to pain and function (Al-Bayati et al., 2018).

Accumulating evidence has demonstrated that patients with either unilateral or bilateral KOA exhibit certain degrees of interlimb asymmetries. A cohort study by Creaby et al. revealed that unilateral KOA patients present with significant asymmetry in knee flexion torque, whereas bilateral KOA patients show symmetric varus-valgus angles of the knee joint (Creaby et al., 2012). In addition, they found the severity of knee pain was closely associated with the asymmetry in knee biomechanical parameters. A meta-analysis focusing on foot features and mechanics in KOA patients indicated that compared with healthy individuals, KOA patients exhibit a more pronounced interlimb difference in pronated foot posture (Almeheyawi et al., 2021). This conclusion is consistent with the results of our previous study, which confirmed that KOA patients exhibit a significantly greater degree of foot posture asymmetry than healthy controls (Chen et al., 2020). In addition, some studies have found that biomechanical asymmetry of the thigh and calf muscles is also more prevalent in KOA patients than in healthy individuals (Lee et al., 2019; Li et al., 2024). However, there are still few reports on the differences between foot posture asymmetry and muscle asymmetry in unilateral and bilateral KOA patients.

The QF is closely related to the progression of KOA. It is composed of the rectus femoris (RF), vastus medialis (VM), vastus lateralis (VL) and vastus intermedius (Shen et al., 2024). QF is the primary muscle group responsible for knee extension and plays a pivotal role in maintaining knee mobility (Petrella et al., 2017). A Cross-sectional study has demonstrated that in patients with unilateral KOA, the adduction strength of QF on the affected side is approximately 16% lower than that on the non-affected side. Furthermore, compared with healthy people, the strength of the affected and non-affected quadriceps femoris in KOA patients is significantly reduced during adduction, abduction, flexion and extension activities (Hislop et al., 2022). When strength imbalances occur within the QF such as strength weakening of the VM, it triggers lateral displacement of the patella and elevates pressure on the patellofemoral joint, thereby leading to patellofemoral osteoarthritis or exacerbating the medial compartment lesion of the knee joint (Siqueira et al., 2022). Meanwhile, some scholars have proposed that the change in foot posture was related to the knee adduction moment and could affect the stability of the knee joint (Solomonow-Avnon et al., 2019), thereby inducing knee osteoarthritis. In addition, in patients with KOA, there may be an interaction between foot posture and changes in QF. On the one hand, abnormal foot posture can disrupt lower limb alignment and increase knee joint instability, which consequently elevates the load on the QF, leading to increased muscle fatigue and reduced strength. On the other hand, when quadriceps strength weakens, it cannot to counteract the mechanical deviations caused by abnormal foot posture, further amplifying the abnormal loads on the knee joint. It exacerbates knee articular degeneration and forms a vicious cycle (Chen et al., 2023).

In KOA patients, abnormal foot postural alignment can cause mechanical imbalance in the limbs, leading to a decline in lower limb muscle function and coordination, which may result in changes in the muscle properties of the QF. These changes in muscle properties can be measured using a non-invasive digital palpation device (MyotonPRO), which can accurately assess the properties of superficial muscles, including muscle tone and stiffness. Surface electromyography (sEMG) can be utilized to measure muscle activation during both static and dynamic movements of the upper and lower limbs (Schmidt et al., 2016). Root mean square (RMS) values reflect the effective firing of neuromuscular fibers and are employed to assess the level of muscle contraction (Gerdle et al., 2000). Considering the interaction among the QF, foot posture, and KOA, we hypothesized that there might be a correlation between foot posture asymmetry and QF muscle properties in KOA patients. Based on the aforementioned equipment and methods, the purpose of this study is to investigate the differences in QF properties, sEMG parameters, and foot posture asymmetries between patients with unilateral and bilateral KOA, and to analyze the factors related to foot posture asymmetry, aiming to provide a theoretical basis for the prevention and treatment of KOA.

## Methods

### Study design

The study was conducted at the Orthopedic Clinic of Guangdong Second Hospital of Traditional Chinese Medicine from August 2023 to February 2024. This study protocol received approval from the Ethics Committee of the Guangdong Second Hospital of Traditional Chinese Medicine (No. 2021(K58)) and has been registered with the Chinese Clinical Trial Registration Center (Registration number: ChiCTR2100050269, Registration Date: 25 August 2021). Prior to their participation in this study, all participants provided written informed consent. All methods were performed in accordance with relevant guidelines and regulations.

### Participants

All participants in this study were enrolled from the outpatient department of orthopedics at the Guangdong Second Traditional Chinese Medicine Hospital. Patients with a confirmed diagnosis of KOA were identified through a review of the hospital’s electronic medical records. Eligible participants were subsequently contacted via telephone for follow-up and invited to participate in a face-to-face interview, at which time informed consent was obtained and baseline assessments were conducted. The inclusion criteria were as follows: (1) Participants aged between 60 and 75 years old, who were diagnosed with KOA in accordance with the American College of Rheumatology clinical criteria; (2) With a BMI ranging from 18 to 25 kg/m^2^; (3) Kellgren/Lawrence (Kellgren and Lawrence, 1957) (K/L) grade ≥1 in unilateral or bilateral knee involvement; (4) Predominant involvement of the medial compartment of KOA; (5) An ability to complete a 15-s straight leg-raising exercise; (6) an ability to stand independently on the platform without any external assistance. Exclusion criteria were as follows: (1) presence of other inflammatory arthritis; (2) presence of nervous system diseases, such as stroke, spinal disease, Parkinson’s disease, serious cardiovascular or respiratory disease, or other musculoskeletal diseases; (3) the knee joint has received treatment in the last 6 months; (4) history of surgery on the knee or other parts of the lower extremity; (5) congenital or traumatic structural deformities of the lower limbs; (6) any medication that affects properties of muscle and tendon; (7) participation in vigorous exercise within 48 h prior to the study. Additionally, our study focused on patients with medial compartment KOA, as its prevalence in China is significantly higher than that of lateral compartment KOA. The sample size was calculated using PASS 15.0.5, and the estimation was based on previous studies regarding the asymmetry percentage of the FPI (Chen et al., 2021). The effect size of the FPI-6 asymmetry index for patients with bilateral KOA and those with unilateral KOA was 0.72. With α set at 5% and power at 80%, the sample size for each group was calculated to be 25 participants. Ultimately, we included 32 patients with unilateral KOA and 35 patients with bilateral KOA, who were divided into the unilateral group (UG) and the bilateral group (BG). We designated the asymptomatic or less symptomatic side as the relatively moderate leg (RML), and the symptomatic or more severe side as the relatively severe leg (RSL).

### Measurement of sEMG (RMS) of the QF

Our research team randomly selected one member (JH-C) to perform measurements using the Noraxon surface electromyography (sEMG) telemetry system (Milan, Italy, model: BTS S.P.A, FreeEMG1000, software version: FreeEMG-3.3.7.0). The members of our research team who conducted this evaluation underwent an 8-week professional training in sEMG telemetry systems and completed professional evaluation tests. The parameters of the Noraxon telemetry system were configured as follows: the channel sampling bandwidth ranged from 20 to 500 Hz, the sensitivity was set at 1 mV, and the EMG data acquisition frequency was set to 1,000 Hz. We utilized six channels for recording sEMG signals from RF, VM, and VL on both sides. Regarding electrode placement selection: (1) RF: electrodes were placed at the midpoint of the line between the superior edge of the patella and the anterior superior iliac spine in front of the thigh; (2) VM: the electrode were placed at 20% of the distance between the medial joint space of the knee and the anterior superior iliac spine, and the angle between the connecting line of the two electrodes and the long axis of the femur was 55°; (3) VL: the electrodes were positioned superior to the patellar angle and at the lateral joint space of the knee, and attached 1/10 of the distance from the iliac spines (with an angle between connecting line of two electrodes and femur’s long axis being 15°). The center distance between the two electrodes was 2 cm (Chen et al., 2023). The measurement was conducted at 25 °C room temperature. Prior to measurement, hair at the test site of each participant was removed. This was followed by disinfecting the skin with a cotton ball soaked in 75% medical alcohol and wiping sweat at and around the electrode site with gauze pads. In previous studies, the sEMG signals of functional activities in patients with KOA included squatting tasks and straight leg raising tasks (Pan et al., 2023; Chen et al., 2023). Considering that some of the KOA patients included in our study had K/L grades of 3 and 4 and were unable to independently complete the 30-s squatting task, our study only collected the sEMG signals of KOA patients during the straight leg raising task. Test procedure (straight leg-raising task): Participants were asked to assume a supine position on the treatment bed, relax the muscles of the lower limb, and extend the knee joint. When recording the sEMG signal, participants were instructed to elevate their lower limb to an angle of 30° above the bed surface for approximately 15 s. Following signal acquisition, the lower limb was returned to its initial position, and the QF on the opposite side was measured using the same methodology. The measurement sequence commenced with the left lower limb followed by the right lower limb. The procedure was repeated three times, with a 15-min interval between each measurement. Processing of surface electromyography data: The EMGAnalyzer2.9.25 analysis software was utilized for the correction, filtering, and smoothing of the raw electromyogram data, as well as for calculating the RMS (Supplementary Figure S1). To minimize errors, RMS values were selected within the range of 2–12 s. The final RMS values were obtained by averaging three repeated measurement periods.

### Evaluation of the mechanical properties of the QF

Our research team randomly selected one member (JQ-T) to use a small, handheld non-invasive MyotonPRO device (MyotonPRO, Estonia, serial number: 000297, product manufacturers code: 1308600502) for assessing the mechanical properties of the QF on both sides. Prior to formal data collection, the researcher was trained in the operating procedures and familiarized with the use of the MyotonPRO. Prior to measurement, participants were asked to rest for 5 min, then assume a supine position on the treatment bed with the knee joint in 0° extension position. The designated positions for measurement were as follows: The RF measurement point was positioned at two-thirds of the distance between the anterior superior iliac spine and the superior edge of the patella. When selecting the measurement points for the VM and VI, participants were asked to actively contract their lower limbs to extend the hip and knee joints, and the muscular bulges near the knee joints on both sides served as the measurement points (Shen et al., 2024). The measurement order was as follows: left RF, right RF, left VM, right VM, left VL, and right VL. Following the measurement of muscle tone and stiffness in one limb, participants were instructed to lie in a supine position and rest for 5–10 min prior to assessment of the contralateral limb. At the start of measurement, participants were instructed to hold their breath for 5 s before the test cycle began, to minimize the impact of confounding factors caused by fluctuations in abdominal pressure (Chen et al., 2019). The operator held MyotonPRO in one hand and placed the equipment probe vertically on the participants’ survey points located on the skin surface (Supplementary Figure S2). When a suitable pressure was applied to the probe, the indicator changed from red to green, indicating the start of the measurement. This was followed by five consecutive vibrations, indicating the completion of the measurement. The instrument could apply a pretension pressure of 0.18 N to slightly compress the subcutaneous superficial tissue and rapidly released a mechanical impulse force of 0.58 N for 15 ms, inducing a naturally damped oscillation in the muscle tissue. Based on the oscillation signal, the frequency (F; Hz) and stiffness (S; N/m) of the oscillation were determined (Shen et al., 2024). Throughout the measurement process, researchers remained unaware of participant group information or which limb was experiencing pain. Following measurement completion, variations coefficient (CV) was observed to assess error magnitude. If the CV exceeded 3%, retesting was required. Muscle tone and stiffness values were averaged across three repeated measurement cycles.

### Evaluation of the foot posture

The reliability and consistency of the FPI-6 for assessing KOA patients have been confirmed in our previous studies (Shen et al., 2024; Wang et al., 2023). Our research group randomly selected one rater (XX-S) to assess the static foot posture of both feet using the FPI-6 score. The rater attended a training session on the FPI-6 and interacted with other team members to familiarize themselves with each item of the assessment tool. During the assessment, participants were asked to stand barefoot in a neutral position, with their arms naturally hanging at their sides and eyes looking straight ahead, to avoid foot posture changes caused by rotation (Shen et al., 2024). The FPI-6 consists of six items: (1) talar head palpation, (2) curves above and below the lateral malleolus, (3) talonavicular joint bulging, (4) calcaneal frontal plane position, (5) medial longitudinal arch height and congruence, (6) forefoot abduction or adduction (Wang et al., 2023). Each item was scored from −2 to 2 points, resulting in a total FPI-6 score ranging from −12 to +12. The classification of FPI-6 scores was as follows: a score ≥10 indicated severe pronation, 6–9 indicated mild pronation, 0–5 indicated neutral alignment, −1 to −4 signified mild supination, and ≤ −5 signified severe supination (Redmond et al., 2006). Throughout the assessment process, the rater was kept blinded to participants’ basic information and painful sites.

### Calculation of the asymmetry indexes and assessment of the FPI posture asymmetry

According to previous research methods (Supplementary Method 1), we calculated the asymmetry indexes of muscle tone, stiffness, and RMS for patients in both groups (Chen et al., 2021). A higher asymmetry index value indicates a more pronounced imbalance in muscle performance between the two sides. The muscle tone asymmetry indexes, stiffness asymmetry indexes and RMS asymmetry indexes were denoted as Asy_Tone_ (RF), Asy_Tone_ (VM), Asy_Tone_ (VL), Asy_Stiffness_ (RF), Asy_Stiffness_ (VM), Asy_Stiffness_ (VL), Asy_RMS_ (RF), Asy_RMS_ (VM), and Asy_RMS_ (VL). In addition, we used the FPI asymmetry score to categorize foot posture asymmetry in both groups. This score was calculated as the FPI score of the right foot minus the FPI score of the left foot (Rokkedal-Lausch et al., 2013). Asymmetry score ranged from −2 to +2 defined as normal, −4 ≤ asymmetry score < −2 or +2 < asymmetry score ≤+4 was defined as asymmetric, and score < −4 or >4 was defined as severely asymmetric.

### Statistical analysis

Statistical analyses were conducted using SPSS 26.0 software (IBM, Corp, NY, United States). The normality of the study’s continuous variables was assessed using the Shapiro-Wilk test. Measurement data with a normal distribution were presented as mean ± standard deviation (SD; x ± s), whereas measurement data with a non-normal distribution were presented as median (interquartile range, IQR). Categorical variables were expressed as percentages and compared using chi-square test. The homogeneity of variance was assessed using Levene’s test. Based on the normality test results, the paired Student’s t-test was used to compare inter-limb differences within each patient group for normally distributed data, whereas the independent samples t-test was used to compare between-group differences. The nonparametric Mann-Whitney U test was used to compare measurement data with a non-normal distribution. Multivariate linear regression analysis was performed to estimate the regression coefficient (unstandardized coefficient: *b*) and its 95% confidence interval (95% CI). Additionally, the association between QF properties asymmetry index, RMS asymmetry index, BMI, VAS, K/L grade and FPI asymmetry index was analyzed, with adjustment for key covariates (gender, age, weight, joint dorsiflexion range). In the multiple linear regression analysis, the foot posture asymmetry index was designated as the dependent variable, and the QF characteristic asymmetry index, RMS asymmetry index, BMI, VAS score, and K/L grade were designated as the independent variables. The level of statistical significance was set at *P* < 0.05.

## Result

### Participants characteristics

A total of 32 patients with unilateral KOA and 35 patients with bilateral KOA were enrolled in this study, according to the predefined inclusion and exclusion criteria. The demographic characteristics and foot posture of all participants are presented in Table 1. No significant differences were observed between the two groups in terms of age, weight, height, BMI, gender, VAS score, or K/L grade.

**TABLE 1:** Baseline and foot posture characteristics of the study participants.

Variables	UG (n = 32)	BG (n = 35)	p value
Age (years)	66.81 ± 4.10	68.54 ± 4.06	0.839
Height (cm)	164.31 ± 5.69	161.74 ± 6.99	0.106
Weight (kg)	60.61 ± 6.17	58.36 ± 6.67	0.157
BMI (kg/m2)	22.45 ± 2.07	22.26 ± 1.52	0.660
Gender (male/female)	8/24	11/24	0.560
VAS (RSL)	6.28 ± 1.75	7.11 ± 1.80	0.059
Kellgren-Lawrence classification			
Grade 1	3	2	—
Grade 2	10	8	—
Grade 3	15	15	—
Grade 4	4	9	—
RSL (left/right)	14/18	16/19	—
RML (left/right)	18/14	19/16	—
FPI score			
Left	3.75 ± 4.24	2.71 ± 2.87	—
Right	3.88 ± 2.85	2.91 ± 3.12	—
RSL	4.19 ± 4.19	3.06 ± 3.28	—
RML	3.44 ± 2.88	2.57 ± 2.66	—
FPI asymmetry score	0.13 ± 3.49	0.20 ± 3.06	—

UG, unilateral group; BG, bilateral group; BMI, body mass index; K/L grade, Kellgren/Lawrence grade; RSL, relatively severe leg; RML, relatively moderate leg; FPI, foot posture index.

### Analysis of QF muscle sEMG (RMS)

**FIGURE 1:**
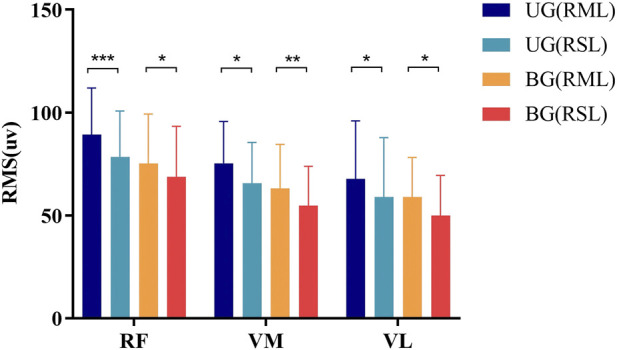
RMS values of the QF during the straight leg-raising Task. UG, unilateral group; BG, bilateral group; RSL, relatively severe leg; RML, relatively moderate leg; RF, rectus femoris; VM, vastus medialis; VL, vastus lateralis; * indicates P < 0.05; ** indicates P < 0.01; *** indicates P < 0.001.

During the straight leg-raising task, the RMS values of the RF, VM, and VL on the RML were significantly higher than those on the RSL in both the BG and UG (*P* < 0.05), as depicted in Figure 1. In addition, during the straight leg-raising task, the Asy_RMS_ (VM) in the UG was significantly higher than that in BG (*P* < 0.01). However, no significant differences were observed in Asy_RMS_ (RF) and Asy_RMS_ (VL) between the UG and BG, as shown in Table 4.

### Analysis of QF muscle tone

The results indicated that the muscle tone of the RF, VM, and VL on the RSL side was significantly higher than that on the RML in both the UG and BG (*P* < 0.05). In the UG, more significant increases in muscle tone were observed for the VM and VL (*P* < 0.001), as shown in Table 2. In addition, significant differences were observed in the lower limb muscle tone asymmetry indexes of the RF, VM, and VL between the two groups. The Asy_Tone_ (RF) and Asy_Tone_ (VM) of the UG were significantly higher than those in the BG (*P* < 0.05), whereas the Asy_Tone_ (VL) of UG was significantly lower than that in the BG (*P* < 0.01), as shown in Table 4.

**TABLE 2:** Comparison of QF muscle tone between the two groups (Hz).

Group side	UG		p value	BG		p value
	RSL	RML		RSL	RML	
RF	14.69 ± 1.56	13.98 ± 1.79	0.017	15.03 ± 1.34	14.57 ± 1.50	0.013
VM	14.51 ± 1.87	13.47 ± 1.67	0.008	15.28 ± 1.70	14.73 ± 1.99	0.030
VL	15.81 ± 1.78	15.23 ± 1.82	0.001	16.02 ± 1.80	15.57 ± 1.83	0.013

UG, unilateral group; BG, bilateral group; RSL, relatively severe leg; RML, relatively moderate leg; RF, rectus femoris; VM, vastus medialis; VL, vastus lateralis.

### Analysis of QF stiffness

The results indicated that the stiffness of the RF, VM, and VL on the RSL of the UG was significantly higher than that on the RML (*P* < 0.05). In the BG, the stiffness of the RF, VM, and VL on the RSL was also significantly higher than that on the RML (*P* < 0.05), as shown in Table 3. In addition, there were significant differences in Asy_Stiffness_ (RF), Asy_Stiffness_ (VM), and Asy_Stiffness_ (VL) between the two groups. The Asy_Stiffness_ (RF), Asy_Stiffness_ (VM), and Asy_Stiffness_ (VL) of the UG was significantly higher than that in the BG (*P* < 0.05), as shown in Table 4.

**TABLE 3:** Comparison of QF stiffness between the two groups (N/m).

Group side	UG		p value	BG		p value
	RSL	RML		RSL	RML	
RF	277.06 ± 30.40	262.56 ± 28.63	0.021	290.09 ± 26.68	274.49 ± 28.34	0.006
VM	260.25 ± 30.77	249.56 ± 27.76	0.014	301.91 ± 27.76	292.49 ± 30.82	0.015
VL	289.94 ± 26.66	278.66 ± 31.03	0.009	321.49 ± 29.59	311.83 ± 30.97	0.012

UG, unilateral group; BG, bilateral group; RSL, relatively severe leg; RML, relatively moderate leg; RF, rectus femoris; VM, vastus medialis; VL, vastus lateralis.

**TABLE 4:** Comparison of asymmetry indexes for QF properties and sEMG parameter (RMS) between two groups.

Variables	UG	BG	p value
Asy_Tone_ (RF)	6.51 (4.34, 14.36)	4.20 (2.13, 7.80)	0.017
Asy_Tone_ (VM)	12.25 (6.35, 17.80)	7.46 (4.38, 11.76)	0.009
Asy_Tone_ (VL)	4.08 (2.80, 6.01)	5.95 (4.26, 8.39)	0.006
Asy_Stiffness_ (RF)	9.82 (4.74, 15.46)	5.80 (2.83, 7.95)	0.003
Asy_Stiffness_ (VM)	5.56 (3.59, 9.43)	4.27 (1.89, 6.08)	0.021
Asy_Stiffness_ (VL)	7.12 (3.15, 10.07)	4.10 (1.74, 5.16)	0.001
Asy_RMS_ (RF)	16.38 (10.59, 23.28)	9.57 (6.98, 17.19)	0.070
Asy_RMS_ (VM)	20.00 (14.29, 36.99)	13.27 (7.61, 20.59)	0.002
Asy_RMS_ (VL)	15.79 (9.66, 35.25)	17.05 (7.69, 23.91)	0.254

UG, unilateral group; BG, bilateral group; Asy_Tone_ (RF), asymmetry index of rectus femoris muscle tone; Asy_Tone_ (VM), asymmetry index of vastus medialis muscle tone; Asy_Tone_ (VL), asymmetry index of vastus lateralis muscle tone; Asy_Stiffness_ (RF), asymmetry index of rectus femoris stiffness; Asy_Stiffness_ (VM), asymmetry index of vastus medialis stiffness; Asy_Stiffness_ (VL), asymmetry index of vastus lateralis stiffness; Asy_RMS_(RF), asymmetry index of rectus femoris RMS; Asy_RMS_(VM), asymmetry index of vastus medialis RMS; Asy_RMS_(VL), asymmetry index of vastus lateralis RMS.

### Analysis of foot posture asymmetry

**FIGURE 2:**
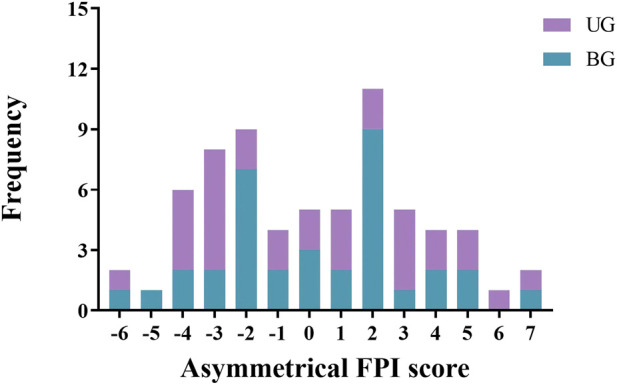
Number of subjects with various FPI asymmetry score distributions in the UG and BG. UG, unilateral group; BG, bilateral group.

According to the FPI asymmetry score, the two groups of patients were categorized into three types: normal, asymmetry, and severe asymmetry. The findings revealed that the percentage of individuals classified as normal in the UG (34.4%) was lower compared with that in the BG (65.7%), whereas the proportion of those categorized as asymmetric (including both asymmetric and severely asymmetric individuals) was higher in the UG (65.6%) than in the BG (34.3%). A statistically significant difference was observed between the two groups (*χ* ^2^ = 6.57, *P* = 0.01), as shown in Table 5. In addition, the majority of asymmetrical FPI scores were concentrated within the range of −2 to 2 in the BG, whereas the UG exhibited a wide distribution of asymmetrical FPI scores, as shown in Figure 2.

**TABLE 5:** Comparison of FPI-6 asymmetry scores between two groups.

Categorical variables	UG	BG	χ^2^value	p value
Normal	11 (34.4%)	23 (65.7%)	—	—
Asymmetry and severe asymmetry	21 (65.6%)*	12 (34.3%)	—	—
Total	32 (100%)	35 (100%)	6.57	0.01

UG, unilateral group; BG, bilateral group. *Compared with the BG, P < 0.01.

### Correlation of QF property asymmetry indexes, RMS asymmetry indexes, BMI, VAS score, and K/L grade with FPI asymmetry index

The results indicated that the VAS score and K/L grade were significantly correlated with the FPI asymmetric score in the UG (*b* = 1.065; 95% CI: 0.194, 1.936; *p* = 0.019 and *b* = 1.770; 95% CI: 0.215, 3.325; *p* = 0.028, respectively), and a similar significant correlation was observed between the VAS score, K/L grade, and FPI asymmetry score in the BG (*b* = 0.665; 95% CI: 0.117, 1.212; *p* = 0.020 and *b* = 1.523; 95% CI: 0.414, 2.632; *p* = 0.009, respectively). No significant correlations were observed between Asy_Tone_ (RF, VM, VL), Asy_Stiffness_ (RF, VM, VL), Asy_RMS_ (RF, VM, VL), BMI, and FPI asymmetry score (*P* > 0.05), as shown in Table 6.

**TABLE 6:** Correlations between QF properties asymmetry indexes, RMS asymmetry indexes, BMI, VAS score and K/L grade with FPI asymmetry index.

Independent variable	*b*	SE	*t* value	*p* value	95% CI
UG (*R* ^2^ = 0.689)					
Asy_Tone_ (RF)	−0.013	0.079	−0.168	0.868	−0.179, 0.152
Asy_Tone_ (VM)	−0.026	0.066	−0.399	0.695	0.165, 0.112
Asy_Tone_ (VL)	0.012	0.223	0.055	0.957	−0.455, 0.479
Asy_Stiffness_ (RF)	0.035	0.082	0.432	0.671	−0.136, 0.207
Asy_Stiffness_ (VM)	−0.145	0.097	−1.495	0.151	−0.349, 0.058
Asy_Stiffness_ (VL)	0.052	0.109	0.477	0.639	−0.177, 0.281
Asy_RMS_ (RF)	0.064	0.066	0.975	0.342	−0.074, 0.203
Asy_RMS_ (VM)	−0.010	0.044	−0.217	0.831	−0.102, 0.083
Asy_RMS_ (VL)	0.030	0.026	1.162	0.260	−0.024, 0.084
BMI	0.125	0.280	0.445	0.661	−0.461, 0.710
VAS score	1.065	0.416	2.560	0.019	0.194, 1.936
K/L grade	1.770	0.743	2.382	0.028	0.215, 3.325
BG (*R* ^2^ = 0.645)					
Asy_Tone_ (RF)	0.045	0.112	0.402	0.692	−0.187, 0.276
Asy_Tone_ (VM)	−0.050	0.096	−0.521	0.608	−0.248, 0.149
Asy_Tone_ (VL)	−0.272	0.194	−1.401	0.175	−0.675, 0.131
Asy_Stiffness_ (RF)	0.096	0.169	0.566	0.577	−0.255, 0.446
Asy_Stiffness_ (VM)	−0.036	0.243	−0.147	0.884	−0.540, 0.469
Asy_Stiffness_ (VL)	−0.194	0.190	−1.022	0.318	−0.587, 0.200
Asy_RMS_ (RF)	0.041	0.051	0.810	0.426	−0.064, 0.146
Asy_RMS_ (VM)	0.066	0.059	1.123	0.273	−0.056, 0.189
Asy_RMS_ (VL)	0.009	0.039	0.230	0.821	−0.071, 0.089
BMI	0.258	0.308	0.837	0.412	−0.381, 0.897
VAS score	0.665	0.264	2.519	0.020	0.117, 1.212
K/L grade	1.523	0.535	2.848	0.009	0.414, 2.632

b, unstandardized coefficient; SE, standard error; 95% CI, 95% confidence interval; UG, unilateral group; BG, bilateral group; Asy_Tone_ (RF), asymmetry index of rectus femoris muscle tone; Asy_Tone_ (VM), asymmetry index of vastus medialis muscle tone; Asy_Tone_ (VL), asymmetry index of vastus lateralis muscle tone; Asy_Stiffness_ (RF), asymmetry index of rectus femoris stiffness; Asy_Stiffness_ (VM), asymmetry index of vastus medialis stiffness; Asy_Stiffness_ (VL), asymmetry index of vastus lateralis stiffness; Asy_RMS_ (RF), asymmetry index of rectus femoris RMS; Asy_RMS_ (VM), asymmetry index of vastus medialis RMS; Asy_RMS_ (VL), asymmetry index of vastus lateralis RMS; BMI, body mass index; K/L grade, Kellgren/Lawrence grade.

## Discussion

The alterations in foot posture and lower limb muscle function observed in patients with KOA may disrupt the biomechanical stability of the knee joint, contribute to limb asymmetry, and thereby exacerbate the progression of KOA. The findings of our study revealed that both unilateral and bilateral KOA patients exhibited significant asymmetries in QF function and foot posture, but the asymmetry degree was more pronounced in the unilateral group. Additionally, VAS score and K/L grade were closely associated with foot posture asymmetry.

### Asymmetry of QF properties and sEMG parameters in the UG and BG

The level of QF activation plays a pivotal role in modulating the load on the knee joint (Segal and Glass, 2011). The RMS value reflects the overall efficacy of muscle response to electrical stimulation and is positively correlated with muscle strength and the degree of muscle contraction. In the straight leg-raising task, our results demonstrated that in both the UG and BG, the RMS of the RF, VM, and VL on the RML was significantly higher than that on the RSL. This finding aligns with the study by Hislop et al. (2022), who reported that KOA patients had lower muscle strength on the more symptomatic side during functional movements. The lower RMS on the RSL may reflect impaired neuromuscular control and muscle fiber recruitment caused by knee pain, as pain can inhibit motor unit firing and reduce muscle contraction efficiency (Gerdle et al., 2000). In addition, research utilizing human CT scans has substantiated that muscle volume on the severely affected side is diminished in patients with KOA, and this reduction correlates with a decline in extensor muscle strength (Tsukada et al., 2020). Mechanistically, the increased proportion of mixed IIa/x fibers and the reduction of satellite cells in KOA patients may significantly drive alterations in muscle activation patterns, and these changes may be associated with QF weakness and pain in KOA (Noehren et al., 2018; Fink et al., 2007; Krishnasamy et al., 2018).

Additionally, we observed that the muscle activation asymmetry of the VM in unilateral KOA patients was significantly higher than that in bilateral KOA patients. This may indicate that patients with unilateral KOA are at a greater risk of VM biomechanical imbalance. VM is a key muscle for patellar stability, and the decrease of its muscle activation ability can disrupt patellofemoral alignment, increase medial compartment pressure, and exacerbate KOA progression (Siqueira et al., 2022). This interlimb asymmetry may be attributed to the following factors: on the one hand, the VM is the last muscle to develop in the QF system and is also the weakest muscle, making it particularly susceptible to disuse atrophy when affected by various factors (Al-Khlaifat et al., 2016). On the other hand, during the progression of KOA, selective atrophy of type II muscle fibers first occurs in the VM (Fink et al., 2007), and the atrophy of type II fibers is associated with pain perception in KOA patients.

Muscle tone refers to the resting tension in muscles or the resistance received by the examiner when assessing muscle relaxation. Abnormally increased muscle tone can impede blood supply through vasoconstriction, thereby exacerbating muscle fatigue (Wu et al., 2021; Ganguly et al., 2021). Muscle stiffness reflects the ability to resist muscle contraction or deformation caused by mechanical forces. An increase in muscle stiffness can lead to a decrease in muscle performance during exercise (Kocur et al., 2019). In our study, we demonstrated that both unilateral and bilateral KOA patients had significantly elevated muscle tone and stiffness in the RSL, which was consistent with findings from our previous study (Shen et al., 2024). In addition, the research of Chang confirmed that VL stiffness in KOA patients was markedly higher than that observed in a healthy population (Chang et al., 2022). Although the exact cause of increased muscle tone and stiffness is not fully understood, this change may be related to age of onset, pain, decreased muscle strength, decreased joint activity, muscle atrophy, and other factors (Gellhorn et al., 2018). As anticipated from the classification of the RML and RSL based on pain levels, prolonged pain could induce alterations in muscle biomechanics. The discomfort experienced within the affected knee joint may function as a protective mechanism to mitigate excessive loading on the joint. Research conducted by Hurwitz et al. (2000) demonstrated a negative correlation between variations in pain levels and changes in knee joint loading. In patients with unilateral KOA, reduced loading on the affected knee joint during walking led to a compensatory increase in loading on the healthy knee joint. Prolonged asymmetrical loading might lead to muscle stiffness and atrophic changes, ultimately leading to muscle weakness and alterations in gait (Kaufman et al., 2001).

Notably, the UG showed more prominent asymmetries in QF mechanical properties: the muscle tone asymmetry indexes of RF and VM, and all stiffness asymmetry indexes of the RF, VM, and VL were significantly higher in the UG than in the BG. Previous studies have indicated a notable limb asymmetry among individuals with unilateral KOA, and this biomechanical imbalance of the knee joint has been closely associated with unilateral pain (Lewek et al., 2006; Creaby et al., 2012). Moreover, in comparison to healthy controls, individuals with unilateral KOA demonstrated diminished knee extension strength, hip strength, and bilateral dynamic balance (Hislop et al., 2022). For unilateral KOA, the RSL bears excessive load, which leads to increased muscle tone and stiffness, making interlimb asymmetry more pronounced. In contrast, for patients with bilateral KOA, the RSL is more likely to transfer part of its load to the less affected the RML through compensation, thereby reducing the overall degree of asymmetry. Given the more pronounced asymmetries in the UG, functional training of QF—especially in the VM, may be considered as one of the rehabilitation programs for KOA patients.

### Foot posture asymmetry and its correlates in KOA

After evaluating the differences in foot posture asymmetry between two groups of KOA patients, we found that both groups had a higher proportion of asymmetric foot posture. This is consistent with a meta-analysis, which found that KOA patients have more pronounced foot posture asymmetry compared to healthy individuals (Almeheyawi et al., 2021). In addition, an observational study involving 50 female patients diagnosed with KOA revealed that the occurrence of foot internal rotation within this group (32%) was significantly higher compared to that observed in healthy controls (4.7%), and the KOA group demonstrated elevated levels of knee and foot pain, along with a higher prevalence of abnormal foot postures (Cigercioglu et al., 2024). Changes in foot posture might have resulted in variations in medial loading on the knee joint, leading to knee pain, a trend that is particularly evident in patients with higher K/L grades (Paterson et al., 2018). Notably, the plantar pressure of patients with KOA increased significantly, and elevated midfoot and central plantar pressure increased the incidence of flat feet and excessive foot pronation in these patients. This may be a factor causing foot asymmetry in KOA patients (Guler et al., 2009; Levinger et al., 2010; F E et al., 2014). Moreover, it was observed that the prevalence of abnormal foot postures among the UG was significantly higher than that in the BG and the distribution of asymmetry scores among the UG exhibited a wider range. Limb asymmetry in patients with unilateral KOA may be associated with alterations in neuromuscular factors, such as muscle strength and proprioception (Shakoor et al., 2011). Reduced muscle strength contributes to decreased fatigue resistance in KOA patients, which in turn leads to increased gait variability and impaired knee joint balance control mechanisms (Helbostad et al., 2007). Moreover, a study revealed that asymptomatic knee joints in patients with unilateral KOA exhibited proprioceptive deficits, which increased the risk of developing bilateral KOA (Sharma, 1999). Another cohort study indicated that 80% of individuals with unilateral KOA progressed to develop KOA in the contralateral knee within 5–12 years (Metcalfe et al., 2012). Therefore, achieving stability in the knee joint requires a combination of various factors, including biomechanics, proprioception, muscle function, and neuromuscular transmission.

We performed multiple linear regression analysis to identify the factors contributing to foot posture asymmetry in patients with KOA. The results revealed significant associations between foot posture asymmetry scores and pain levels as well as K/L grading, regardless of whether the KOA was unilateral or bilateral. Previous studies have demonstrated a positive correlation between knee pain and knee dynamic loading in patients with severe KOA (Henriksen et al., 2012). Additionally, a cross-sectional study conducted to assess foot posture parameters between patients with unilateral and bilateral KOA found that multiple gait parameters, plantar pressure parameters, and asymmetry indices were closely associated with clinical symptoms and disease severity (Wang et al., 2024). Abnormal foot postures can disrupt the normal biomechanical balance of the knee joint, accelerate cartilage degradation and osteophyte formation, and ultimately trigger knee pain and exacerbate disease progression in KOA patients. Given the close association between foot posture and knee joint function, correcting such asymmetry could potentially serve as a therapeutic strategy for the management of KOA. Currently, foot orthotic intervention using insoles has emerged as a reliable approach for the prevention and treatment of KOA. This intervention works by moderately reducing the knee varus angle and external knee torque while slightly increasing ankle valgus, thereby addressing abnormal foot postures (Shaw et al., 2018).

However, our findings did not reveal any significant correlation between QF muscle tone index, stiffness asymmetry index, or RMS asymmetry index of the QF concerning foot posture. This contradicts our initial hypothesis and may be explained by some factors. First, QF may influence foot posture indirectly through knee stability rather than direct alignment control. Second, the interaction between QF function and foot posture may be affected by other unmeasured variables such as ankle muscle strength and hip abductor function (Hislop et al., 2022). Third, the small sample size and inconsistencies in researchers’ interpretation of the FPI-6 may have contributed to the inconsistency in study results. Therefore, the relationship between QF properties, sEMG parameters, and foot posture asymmetry required further research to confirm.

### Limitations

There are several limitations in our study. Firstly, the sample size was inadequate as it solely comprised individuals diagnosed with KOA. It remains uncertain whether there are distinctions in the muscle mechanical properties and activation of the QF between healthy individuals and those with KOA. In future research, our team intends to increase the sample size to conduct a comprehensive analysis of these differences. Secondly, considering that medial compartment KOA is the most prevalent type in China, our study exclusively included patients with this subtype, which may limit the generalizability of our findings. In future, we will incorporate a broader range of KOA subtypes to enhance the reliability of our research outcomes. Lastly, this study solely used the static, subjective FPI-6 to assess foot posture, without validation using dynamic and objective gait measurement tools. Although FPI-6 has been extensively demonstrated as a reliable tool for evaluating foot posture, potential biases may exist. In future research, we intend to integrate the FPI-6 index with gait analysis tools in order to comprehensively and accurately analyze changes in foot posture among KOA patients.

## Conclusion

Both unilateral and bilateral patients with KOA exhibited a propensity for asymmetry in the properties of the QF, RMS values, and foot postures on both sides. Notably, unilateral patients tended to demonstrate this asymmetry more prominently and display a higher prevalence of foot posture asymmetry compared to bilateral KOA patients. Furthermore, the degree of foot posture asymmetry was closely linked to pain severity and K/L grading whether in unilateral or bilateral KOA patients. Therefore, the changes in the muscle properties and sEMG amplitude of the QF as well as the differences in foot posture should be fully taken into account when considering prevention and treatment strategies for KOA.

## Data Availability

The original contributions presented in the study are included in the article/Supplementary Material, further inquiries can be directed to the corresponding author.
