# Supplementary material for: Comparison of quadriceps femoris properties, surface electromyography parameters and foot posture asymmetries between patients with unilateral and bilateral knee osteoarthritis
Source: Front Physiol. 2025 Dec 2;16:1710819. doi: 10.3389/fphys.2025.1710819 (PMC12706165; doi:10.3389/fphys.2025.1710819)
Supplement: Supplementary file 1 [file DataSheet1.docx]

**Supplemental Appendix S1**

**Method 1**

$Asy(\%)=\left( \frac{V_{larger}-V_{lower}}{V_{larger}} \right)\times100$

The muscle tone and stiffness asymmetry index of the upper trapezius muscle was calculated using this equation.

*Asy:* asymmetery index; *V_larger_*: larger value; *V_lower_*: lower value.

**Supplementary Figure 1**


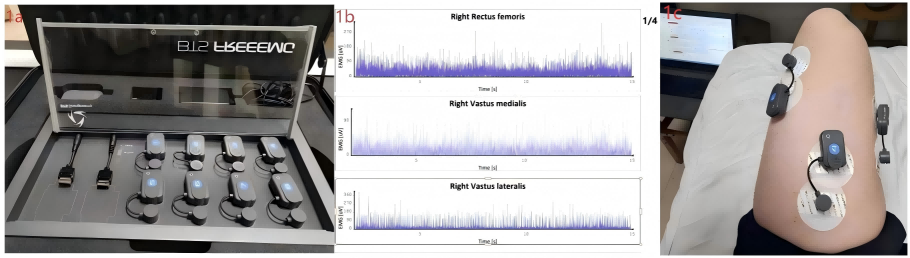


1a: The surface electromyography telemetry system (Italy, model: BTS S. P. A, FreeEMG1000, software version:FreeEMG-3.3.7.0)

1b: The acquisition, interception, filtering, smoothing and other processing of the raw surface electromyography signals.

1c:The measurement position of the quadriceps femoris with surface electromyography.

**Supplementary Figure 2**


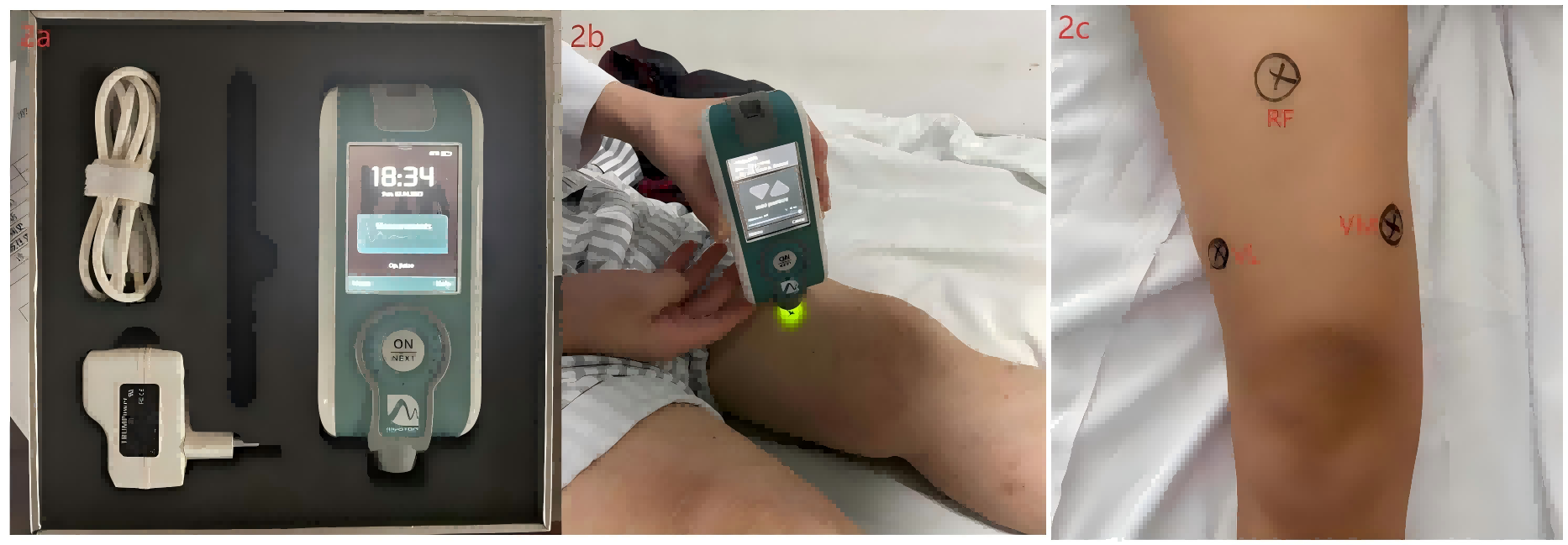


2a: New non-invasive muscle palpation device -MyotonPRO

2b: The researcher employed MyotonPRO to conduct perform measurements.

2c: The measurement position of the quadriceps femoris with MyotonPRO
